# The Slow Component of Oxygen Uptake and Efficiency in Resistance Exercises: A Comparison With Endurance Exercises

**DOI:** 10.3389/fphys.2019.00357

**Published:** 2019-03-28

**Authors:** Manuel V. Garnacho-Castaño, Lluis Albesa-Albiol, Noemí Serra-Payá, Manuel Gomis Bataller, Raquel Felíu-Ruano, Lluis Guirao Cano, Eulogio Pleguezuelos Cobo, José Luis Maté-Muñoz

**Affiliations:** ^1^GRI-AFIRS, School of Health Sciences, TecnoCampus-Pompeu Fabra University, Mataró, Spain; ^2^Department of Rehabilitation, Hospital Asepeyo, Barcelona, Spain; ^3^Department of Physical and Rehabilitation Medicine, Hospital de Mataró, Mataró, Spain; ^4^Department of Physical Activity and Sports Science, Alfonso X El Sabio University, Madrid, Spain

**Keywords:** oxygen uptake kinetics, gross efficiency, energy expenditure, lactate threshold, mechanical fatigue

## Abstract

**Introduction:**

There is a lack of information regarding the slow component of oxygen uptake (VO_2_sc) and efficiency/economy in resistance exercises despite the crucial role played in endurance performance.

**Purpose:**

this study aimed to compare the VO_2_sc, efficiency/economy, metabolic, cardiorespiratory responses, rating of perceived effort and mechanical fatigue between cycling and half-squat (HS) exercises during a constant-load test at lactate threshold (LT_1_) intensity.

**Methods:**

Twenty-one healthy men were randomly assigned in a crossover design to perform cycle-ergometer or HS tests. The order of the two cycle ergometer tests was an incremental test for determining load-intensity in watts (W) at LT_1_, followed by a constant-load test at the LT_1_ intensity. For the three HS tests, the order was a 1RM test to determine the load (kg) corresponding to the 1RM percentages to be used during the second test, incremental HS exercise to establish the load (kg) at the LT_1_ intensity, and finally, a constant-load HS test at the LT_1_ intensity. A rest period of 48 h between each test was established. During the HS and cycle-ergometer constant-load tests, cardiorespiratory and metabolic responses were recorded. Lower limbs fatigue was determined by a jump test before and after the constant-load tests.

**Results:**

A significant exercise mode × time interaction effect was detected in VO_2_, heart rate, energy expenditure (EE), gross efficiency (GE), and economy (*p* < 0.05). A significant and sustained VO_2_ raise was confirmed in HS exercise (*p* < 0.05) and a steady-state VO_2_ was revealed in cycle-ergometer. A higher GE and economy were obtained in HS test than in cycle-ergometer exercise (*p* < 0.001). In both exercises, a non-significant decrease was observed in GE and economy (*p* > 0.05). Lower limbs fatigue was only detected after constant-load HS test.

**Conclusion:**

Although the VO_2_, heart rate and EE responses were higher in cycling exercise, the constant-load HS test induced a greater VO_2_sc and EE raise than the cycling test in a predominantly aerobic metabolism. These results could explain a decrease observed in jump performance only after HS test. GE and economy could benefit from the eccentric phase of the HS exercise.

## Introduction

Laboratory testing of respiratory exchange using a breath-by-breath open-circuit gas analyzer have become a fundamental practice for measuring oxygen uptake (VO_2_) kinetics during constant-load endurance exercises. Pulmonary VO_2_ tends to rise slowly for a given power output beyond ∼3 min during prolonged constant-load endurance exercise, involving sustained lactic acidosis; this surpasses the primary component initiated at exercise onset. This ventilatory phenomenon is known as the slow component of VO_2_ (VO_2_sc) (Gaesser and Poole, 1996).

As some authors suggest, the VO_2_sc could be affected by the behavior of various parameters such as the power-load, VO_2_, and lactate threshold (LT), conditioning cardiorespiratory performance and efficiency (Burnley and Jones, 2007). The power output developed above, below or at the LT will determine the amplitude of the VO_2_sc response. Therefore, LT intensity plays a key role in the assessment of VO_2_sc. According to three-phase model (Skinner and McLellan, 1980), two LTs (LT_1_ and LT_2_) are recognized during cardiopulmonary exercise testing (Binder et al., 2008). LT_1_ is considered as “aerobic threshold” at 40–60% of VO_2max_ (light exercise), and LT_2_ is discerned as “anaerobic threshold” at 60–90% of VO_2max_ (moderate to heavy exercise). Obviously, the VO_2_sc at LT_2_ intensity will increase to a greater extent than at LT_1_ intensity during constant-load exercise.

Despite the important role of VO_2_sc in endurance performance (Lucía et al., 2002), respiratory exchange tests for evaluating power output or VO_2_ at the LT_1_ intensity are not usually applied to resistance exercises in laboratory conditions and, therefore, there is a surprising lack information about VO_2_sc. To date, only one recent study has focused on VO_2_sc in resistance exercises at the LT_1_ intensity (Garnacho-Castaño et al., 2018a). Two findings of this study draw the attention. Firstly, the authors reported a slightly higher VO_2_sc in absolute values (153.8 mL.min^-1^), during 31 min of constant-load HS testing at the LT_1_ intensity in healthy young practitioners, compared to that reported in another study with professional cyclists (130 mL.min^-1^) during 20 min of constant-load test at an intensity above LT_1_ (80% VO_2max_) (Lucía et al., 2000). This detected response of VO_2_sc usually occurs at intensities above the LT_1_ in endurance exercises (Burnley and Jones, 2007). It could be that the VO_2_sc in HS exercise, in a mainly aerobic metabolism (LT_1_), is comparable to the VO_2_sc observed in endurance exercises at intensities above the LT_1_. It has been shown that VO_2_sc is lower in a leg cycle compared to an arm crank exercise (Koppo et al., 2002) and higher in cycling than in running exercise (Billat et al., 1998). This difference between the exercise modes was chiefly associated with the amplitude of response (Carter et al., 2000), which in turn was conditioned by loading intensity during constant-load test (Carter et al., 2002). So, the VO_2_sc is exercise- and intensity-dependent.

Secondly, the authors demonstrated that the continuous increase in VO_2_ and energy expenditure (EE) was linked to a decrease in gross efficiency (GE) (Garnacho-Castaño et al., 2018a). In addition, lower limbs fatigue was detected after constant-load HS test. The VO_2_sc could be explained, at least partly, by the variation in GE which assesses the effects of blood alkalinization on the gradual loss of muscle efficiency (Gaesser and Poole, 1996) and progressive fatigue (Garnacho-Castaño et al., 2018a).

Keeping these two premises in mind, it appears reasonable to suggest a greater increase in VO_2_sc and EE, whereas the efficiency decrease, during HS exercise than during a constant-load cycling test at the LT_1_ intensity. In theory, the power output or load equivalent to the LT_1_ intensity means the highest power output or load that will not elicit VO_2_sc (Burnley and Jones, 2007) during constant-load endurance tests. However, to the best of our knowledge, no studies have compared VO_2_sc, GE, EE, and mechanical fatigue between resistance and endurance exercises during long-term constant-load test at the same aerobic metabolic intensity (LT_1_).

To compare VO_2_sc and efficiency between resistance and endurance exercises could provide relevant information for clarifying the underlying physiological mechanisms that related VO_2_sc and EE to efficiency and fatigue in resistance exercise and, therefore, to determine whether resistance or endurance exercises are more efficient in a predominantly aerobic metabolism. This study aimed to compare VO_2_sc, efficiency/economy, metabolic responses and mechanical fatigue between cycling and HS exercises during a constant-load test at an intensity corresponding to LT_1_.

## Materials and Methods

### Participants

Twenty-one healthy participants were recruited among the male students of the Physical Activity and Sports Sciences Department (age: 21.4 ± 1.5 years, height: 180.2 ± 5.4 cm, weight: 81.8 ± 8.6 kg, body mass index: 25.2 ± 2.0). All participants had at least 6 months of experience in resistance training and were accustomed to HS exercise.

Four exclusion criteria were established: (1) any cardiovascular, metabolic, neurological, pulmonary or orthopedic disorders that could limit exercise performance, (2) the use of any medication, supplements, or performance-enhancing drugs, (3) a one-repetition maximum (1 RM) of less than or equal to 150 kg in HS exercise, (4) being an elite athlete.

Eligible participants were informed of the tests they would be taking, and provided their signed written consent to participate. The participants were instructed to refrain from other exercises or resistance training during the course of the study. The study protocol adhered to the tenets of the Declaration of Helsinki and was approved by the Ethics Committee of TecnoCampus-Pompeu Fabra University (Mataró, Barcelona, Spain).

### Experimental Design

Subjects were required to visit the laboratory on five occasions at the same time each day under similar environmental conditions (temperature 21–25°C, atmospheric pressure 715–730 mm Hg, relative humidity ∼45%). The protocols were implemented according to the procedures previously established by our research group (Garnacho-Castaño et al., 2015a). Participants were randomly assigned in a crossover design to perform cycle ergometer or HS tests.

The order of the two cycle ergometer tests was an incremental test for determining load-intensity in watts (W) at LT_1_, followed by a constant-load test at the LT_1_ intensity. For the three HS tests, the order was a 1RM test to determine the load (kg) corresponding to the 1RM percentages to be used during the second test, incremental HS exercise to establish the load (kg) at the LT_1_ intensity, and finally, a constant-load HS test at the LT_1_ intensity.

A rest period of 48 h between each test was established. During the HS and cycle ergometer constant-load tests, acute cardiorespiratory and metabolic responses were recorded. Timing of blood lactate sampling was the same in both tests. Before and after the constant-load tests, mechanical fatigue in the lower limbs was determined by a counter movement jump (CMJ) test.

### Cycle Ergometry Tests

Incremental and constant-load cycle ergometer tests included a 5-min warm-up on a cycle ergometer (Monark ergomedic 828E, Vansbro, Sweden) at an initial pedaling cadence of 50 rev.min^-1^ and work rate of 50 W, followed by 5 min of dynamic joint mobility drills and stretching exercises. After 2-min rest time, the cycle ergometer tests commenced. In both tests, blood lactate concentrations were measured using a portable lactate analyzer (Lactate Pro LT-1710, Arkray Factory Inc., KDK Corporation, Siga, Japan). The reliability of this device has been previously evaluated (McNaughton et al., 2002).

The incremental test was carried out in a ramp protocol starting with a load of 50 W, which was increased in steps of 25 W.min^-1^ until completing 8 min at a pedaling cadence of 50 rev.min^-1^. Blood samples (5 μL) were attained by finger pricking at rest and every 2 min during the incremental test. The LT_1_ was determined according to three-phase model (Skinner and McLellan, 1980), following the guidelines established by Binder et al. (2008). The LT_1_ was detected by inspecting blood lactate concentrations plotted against workload according to the protocol described by Weltman et al. (1990). The LT_1_ was defined as the highest exercise load completed when a 0.5 mmol.L^-1^ rise over baseline is detected in at least 2 instances.

The constant-load cycle ergometer test involved continuous pedaling at a cadence around 70–80 rev.min^-1^ at an intensity (W) equivalent to the LT_1_ previously determined in the incremental test. The test duration was 31 min. Blood lactate samples were obtained at the start of the test and at the following minutes of cycling: min 4, min 8.5, min 13, min 17.5, min 22, min 26.5, and min 31. Respiratory exchange data were recorded during the constant-load test using a breath-by-breath open-circuit gas analyzer (Vmax spectra 29, Sensormedics Corp., Yorba Linda, CA, United States), which had been previously calibrated. VO_2_, minute ventilation (VE), carbon dioxide production (VCO_2_) and respiratory exchange ratio (RER) were monitored. Heart rate was checked every 5 s by telemetry (RS-800CX, Polar Electro OY, Finland).

### Half Squat Tests

In HS tests, a Smith machine (Matrix Fitness, Johnson Health Tech, Cottage Grove, MN, United States) was used to ensure controlled movements. Each HS test started with a warm-up consisting of 5 min of low intensity running and 5 min of joint mobility. This was followed by a specific warm-up consisting of 1 set of 3–5 repetitions (HS) at a relative intensity of 40–60% of the maximum perceived effort. After 2-min, HS test protocols commenced.

Establishing the 1RM involved 3–5 lifting attempts using increasing weight. The 1RM was defined as the last load lifted by the subject, completing a knee extension to the required position. The rest period between each attempt was 4 min (Garnacho-Castaño et al., 2018a).

The incremental HS test was carried out in 5 sets at relative intensities of 10, 20, 25, 30, 35, and 40% 1RM as previously described (de Sousa et al., 2012; Garnacho-Castaño et al., 2015a,b, 2018a). Each set lasted 1 min and involved 30 repetitions of 2 s each (1 s for both eccentric and concentric muscle actions). This rhythm was checked with a metronome while a researcher provided visual and verbal cues. A passive rest period of 2 min between sets (Garnacho-Castaño et al., 2015a,b) was provided while blood samples were collected for LT_1_ and the load was augmented. The test was terminated voluntarily by the participant or when he was powerless to continue performing repetitions at the set cadence or did not correctly execute repetitions. Blood samples (5 μL) were obtained by finger pricking 30 s after the end of each set, and lactate levels were measured using the same portable lactate analyzer.

The LT_1_ was recognized by means of the algorithm adjustment method based on Orr et al. (1982) as the load-intensity at which blood lactate concentrations start to increase in an exponential manner (Wasserman and McIlroy, 1964). The LT_1_ was detected through computerized 2-segment linear regression by fixing the 2 linear regression equations emerging for each segment at the point of intersection between a plot of blood lactate concentration and relative intensity. Data analysis was performed using Matlab version 7.4 (MathWorks, Natick, MA, United States).

The constant-load HS test was conducted as 21 sets of 15 repetitions of 2 s each (1 s for both eccentric and concentric phases) guided by metronome, visual, and verbal cues. The duration of each set was 30 s and the rest period between sets was 1 min. These guidelines were established in preliminary trials and, subsequently, in previous studies (Garnacho-Castaño et al., 2015a,b). In the constant-load test, it was not possible to perform HS sets in a time longer than 30 s. Furthermore, a recovery period of less than 60 s between sets could not be standardized because in both cases the blood lactate concentrations increased exponentially.

The whole constant-load test took 31 min. Respiratory exchange and heart rate data were recorded as previously described (Garnacho-Castaño et al., 2015a,b). Blood samples were obtained at rest and 30 s after the end of the HS sets (S) S3, S6, S9, S12, S15, S18, and S21, when lactate concentrations were obtained as described above for the incremental test.

### VO_2_ Slow Component, Efficiency/Economy in Constant-Load Tests

In both HS and cycle constant-load tests, the VO_2_sc was identified as the difference between end-of-exercise VO_2_ and the VO_2_ at the end of the third minute of constant-load exercise (Δ VO_2_, in mL.min^-1^). The latter was taken as the average VO_2_ from 2 min 30 s to 3 min 30 s (set 2 to set 3); end-exercise values were taken as the average of the last 2 min of the tests (29 min 0 s to 31 min 0 s, set 20 to set 21). Mean cycling- (CE) and HS-economy (HSE) was expressed in W.L^-1^.min^-1^. GE was calculated as the ratio of work accomplished per minute (i.e., W in kcal.min^-1^) to energy consumed per minute (i.e., in kcal.min^-1^) as follows:

GE (%)=(Work accomplished/EE)×100.

The mean power output during the same period as the respiratory exchange collection was recorded in order to determine “Work accomplished,” which was converted into kcal.min^-1^ as follows:

$$\mathrm{Work}\;\mathrm{accomplished}\;(\mathrm{kcal}.\min^{-1})=\mathrm{Power}\;\mathrm{output}\;(W)\times 0.01433.$$

Energy expenditure was calculated from VO_2_ and the RER. The calorific equivalent of O_2_ was determined from the corresponding RER, using the tables provided by Peronnet and Massicotte (1991).

$$\mathrm{EE}\;(\mathrm{kcal}.\min^{-1})={\mathrm{VO}}_{2}\;(L.\min^{-1})\;\times \;\mathrm{Kcal}.L^{-1}\;\mathrm{of}\;O_{2}.$$

The power output to quantify HSE and GE during HS test was calculated by means of a reliable and validated linear position transducer (Tendo Weight-lifting Analyzer System, Trenèín, Slovakia) (Garnacho-Castaño et al., 2015c). The power output was computed in each repetition based on bar velocity (Lake et al., 2012). The mean power output was calculated as the mean of all repetitions.

### Lower Limbs Mechanical Fatigue

Lower limbs fatigue was evaluated in a CMJ test using a force plate (Quattro Jump model 9290AD; Kistler Instruments, Winterthur, Switzerland), as previously described (Garnacho-Castaño et al., 2015a,b). Jump height, mean power, and peak power were recorded before the start and at the end of both constant-load tests, immediately after the last blood lactate reading. Participants carried out 3 jumps and the mean height, mean power, and peak power output were used in the data analysis. A recovery period of 30 s between each jump was established.

### Perceived Effort

The Borg scale was used to monitor the rating of perceived effort (RPE) (Borg, 1978). Scores were recorded by each subject at the blood collection time points for blood lactate determination during incremental and the constant-load tests.

### Statistical Analysis

The Shapiro–Wilk test was used to check the normal distribution of data, provided as means, standard deviation (SD), confidence intervals (95% CI) and percentages. To identify significant differences between HS and cycle ergometer exercises in VO_2_ kinetics, lactate levels, and economy/efficiency variables during constant-load tests, a general linear model with a two-way analysis of variance (ANOVA) for repeated measures was performed. The two factors were exercise mode (HS and cycle ergometer) and time (corresponding to 7 checkpoints performed in both tests). When appropriate, a Bonferroni *post hoc* adjustment for multiple comparisons was implemented. To determine mechanical fatigue, an ANOVA for repeated measures was performed. A Student’s *t*-test was used to compare heart rate, VO_2_, RPE and blood lactate concentrations at LT_1_ intensity during incremental test in cycle ergometer and HS exercises.

Partial eta-squared ($\eta_{p}^{2}$) was computed to determine the magnitude of the response to both exercise modes. The statistical power (SP) was also calculated. Intraclass correlation coefficients and coefficients of variation percentage were used to determine the relative and absolute reliability. All statistical methods were performed using the software package SPSS Statistics version 23.0 for Mackintosh (SPSS, Chicago, IL, United States). Significance was set at *p* < 0.05.

## Results

Descriptive data related to incremental-load test in cycle ergometer and HS exercises are presented in Table 1. Differences in VO_2_, heart rate, metabolic, RPE and economy/efficiency responses between HS vs. cycle ergometer during constant-load tests are shown in Table 2. Mean intraclass correlation coefficient and mean coefficient of variation for all VO_2_, metabolic and economy/efficiency variables was 0.982 (0.966–0.991) and 5 ± 2%, respectively.

**Table 1:** Data related to 1RM- and incremental-load tests.

Variables	HS	CYC
1RM (Kg)	200.3 (39.7)	–
HS load at LT_1_ (kg)	49.6 (16.2)	–
Relative intensity at LT_1_ (%1RM)	23.9 (4.8)	–
Load at LT_1_ (W)^∗^	242.6 (86.9)	168.1 (38.2)
VO_2_ at LT_1_ (mL.kg^-1^.min^-1^)^δ^	2.08 (0.32)	1.96 (0.37)
Lactate at LT_1_ (mmol.L^-1^)^δ^	2.51 (0.59)	2.21 (0.51)
HR (beats.min^-1^)^δ^	134.95 (16.84)	125.43 (17.16)
HR (%)^δ^	63.14 (8.53)	67.96 (8.60)
RPE (6–20)^δ^	10.62 (1.80)	9.81 (2.09)


*Data are presented as mean and standard deviation (SD). 1RM, one-repetition maximum; CYC, cycle-ergometer; HR, heart rate; HR (%), percentage regards theoretical maximum heart rate; HS, half-squat; LT_*1*_, lactate threshold one; RPE, rating of perceived exertion; VO_*2*_, oxygen uptake. ^∗^Significant differences between HS and cycle ergometer. ^δ^No significant differences between HS and cycle ergometer.*

**Table 2:** Differences in VO_2_, heart rate, RPE, metabolic and economy/efficiency responses between half-squat vs. cycle-ergometer during constant-load test at LT1 intensity.

	HS (95% CI)	CYC (95% CI)	*P^*1*^* ES/SP	*P^*2*^* ES/SP	*P^*3*^* ES/SP
VO_2_ (L.min^-1^)	1.60	2.26	**<0.001**	**<0.001**	**0.001**
	(1.51–1.68)	(2.06–2.46)	0.56/1.00	0.64/1.00	0.17/0.97
HR (beats.min^-1^)	125.91	143.58	**<0.001**	**<0.001**	**<0.001**
	(119.40–132.41)	(134.85–152.31)	0.63/1.00	0.60/0.99	0.29/1.00
RER	0.94	0.92	**<0.001**	0.224	0.879
	(0.92–0.95)	(0.90–0.94)	0.44/1.00	0.07/0.22	0.02/0.16
Lactate (mmol.L^-1^)	3.04	2.92	0.670	0.581	0.168
	(2.69–3.39)	(2.35–3.48)	0.03/0.26	0.02/0.08	0.07/0.58
VO_2_sc (L.min^-1^) (at each checkpoint)	0.09	0.05	0.10	**<0.001**	**0.03**
	(0.05–0.12)	(0.03–0.08)	0.13/0.37	0.35/1.00	0.11/0.82
EE (Kcal.min^-1^)	7.93	11.19	**<0.001**	**<0.001**	**0.001**
	(7.48–8.37)	(10.19–12.19)	0.58/1.00	0.63/1.00	0.17/0.97
GE (%)	43.49	17.66	**<0.001**	**<0.001**	**<0.005**
	(37.65–49.33)	(15.62–19.69)	0.53/1.00	0.75/1.00	0.14/0.92
EC (W.L^-1^.min^-1^)	150.78	60.91	**<0.001**	**<0.001**	**<0.013**
	(130.46–171.11)	(69.07–86.73)	0.49/1.00	0.76/1.00	0.13/0.88
RPE	9.93	10.49	**<0.001**	0.141	0.92
	(9.11–10.76)	(9.65–11.33)	0.622/1.00	0.11/0.31	0.17/0.14


*CYC, cycle-ergometer; EC, economy; EE, energy expended; ES, effect size; GE, gross efficiency; HR, heart rate; HS, half-squat; L, liter; LT1, lactate threshold one; min, minute; RER, respiratory exchange ratio; RPE, rating of perceived exertion; SP, statistical power; VO_*2*_, oxygen uptake; VO_*2*_sc, slow component of oxygen uptake; W, watt. *P^*1*^* Significant differences for time effect. *P^*2*^* Significant differences for exercise mode effect. *P^*3*^* Significant differences for exercise mode × time interaction effect. Data are provided as mean and 95% confidence intervals (95% CI).*

### VO_2_, Lactate, RPE and Heart Rate Responses at LT_1_ During Incremental Tests

No significance differences were found between cycle ergometer and HS exercises in VO_2_, lactate, RPE and heart rate responses at LT_1_ during incremental tests (*p* > 0.05).

### VO_2_, Heart Rate, Respiratory Exchange Ratio, Lactate and RPE During Constant-Load Tests

**FIGURE 1:**
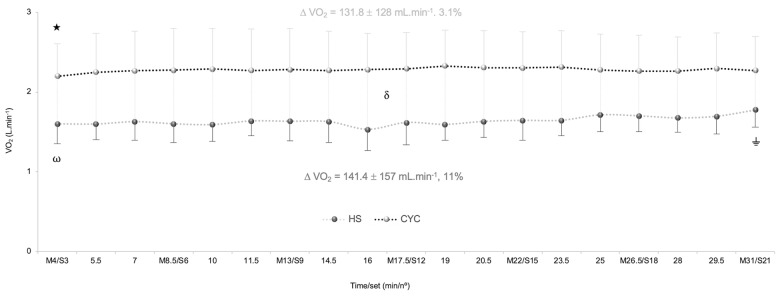
Differences in slow component of oxygen uptake (VO_2_sc) between half-squat (HS) exercise and cycle ergometer (CYC) during constant-load test. ω Significantly different from S6 (*p* = 0.027), S18 (*p* = 0.001), and S21 (*p* = 0.001). 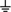 Significantly different from S3 (*p* = 0.001), S6 (*p* = 0.043), and S12 (*p* = 0.003). 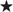 Significantly different from M8.5, M13, M17.5, M22, M26.5, and M31 (*p* < 0.001). δ Significant differences between cycle ergometer and HS exercise at each checkpoint (*p* < 0.001).

In VO_2_, a significant exercise mode x time interaction effect was observed [*p* = 0.001, *F*_(6,120)_ = 4.05, $\eta_{p}^{2}$ = 0.17, SP = 0.97]. A significant time effect [*p* < 0.001, *F*_(6,120)_ = 25.06, $\eta_{p}^{2}$ = 0.56, SP = 1.00], and exercise mode effect were detected [*p* < 0.001, *F*_(1,20)_ = 35.14,$\eta_{p}^{2}$ = 0.64, SP = 1.00]. After Bonferroni adjustment of multiple comparisons, a significant and sustained VO_2_ raise was confirmed from S3 in HS exercise (*p* < 0.05) and a steady-state pulmonary VO_2_ was revealed from M4 in cycle ergometer. Higher VO_2_ was found in cycle ergometer than HS exercise at each checkpoint (*p* < 0.001) (Figure 1).

**FIGURE 2:**
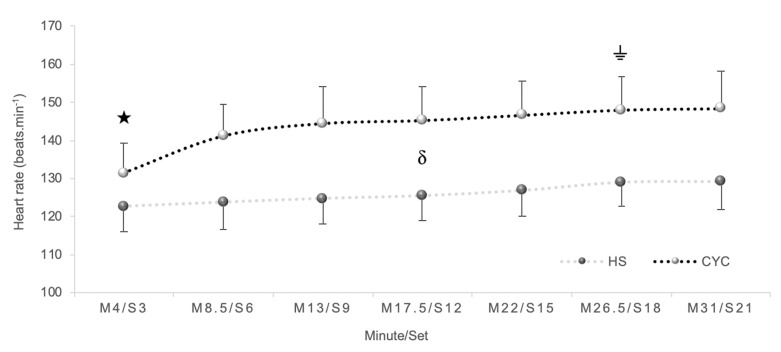
Differences in heart rate between half-squat (HS) exercise and cycle ergometer (CYC) during constant-load test. 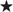 Significantly different from M8.5, M13, M17.5, M22, M26.5, and M31 (*p* < 0.001). 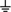 Significantly different from M3, M8.5, M17.5, and M22 (*p* = 0.05). δ Significant differences between cycle ergometer and HS exercise at each checkpoint (*p* < 0.01).

In heart rate, a significant exercise mode x time interaction effect was detected [*p* < 0.001, *F*_(6,120)_ = 8.30, $\eta_{p}^{2}$ = 0.29, SP = 1.00]. A significant time effect [*p* < 0.001, *F*_(6,120)_ = 34.69, $\eta_{p}^{2}$ = 0.63, SP = 1.00], and exercise mode effect were detected [*p* < 0.001, *F*_(1,20)_ = 30.14, $\eta_{p}^{2}$ = 0.60, SP = 0.99]. Bonferroni test determined a higher heart rate in cycle ergometer than HS exercise at each checkpoint (*p* < 0.001) (Figure 2).

**FIGURE 3:**
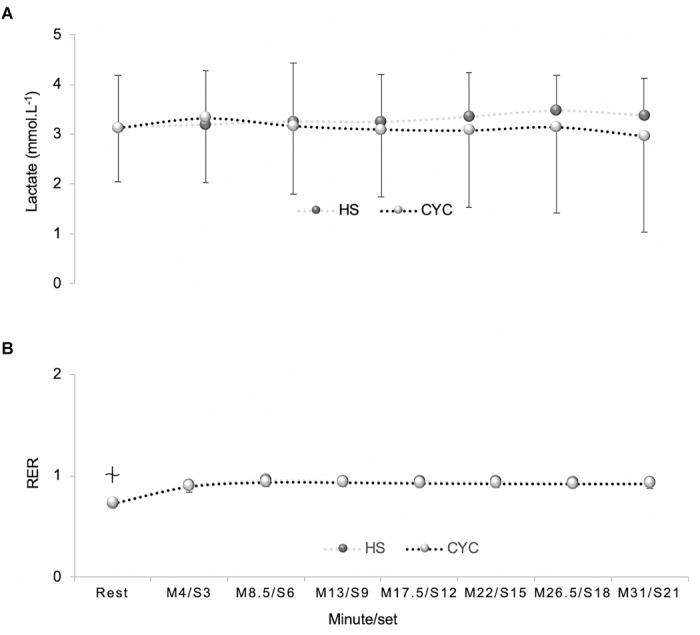
Differences between half-squat (HS) exercise and cycle ergometer (CYC) during constant-load test in: **(A)** Blood lactate. **(B)** Respiratory exchange ratio (RER). No significant differences between cycle ergometer and HS exercises (*p* > 0.05). 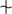 Significantly different from M8.5, M13, M17.5, M22, and M26.5 in cycle ergometer (*p* < 0.05) and significantly different from M8.5, M13, and M17.5 in HS exercise (*p* < 0.01).

No significant exercise mode x time interaction effects or time and exercise mode effects were detected in lactate concentrations (*p* > 0.05) (Figure 3A). It was only detected a time effect in RER [*p* < 0.001, *F*_(6,120)_ = 15.89, $\eta_{p}^{2}$ = 0.44, SP = 1.00] (Figure 3B) and RPE [*p* < 0.001, *F*_(6,120)_ = 32.88, $\eta_{p}^{2}$ = 0.62, SP = 1.00].

### VO_2_sc, Energy Expenditure, Gross Efficiency and Economy During Constant-Load Tests

**FIGURE 4:**
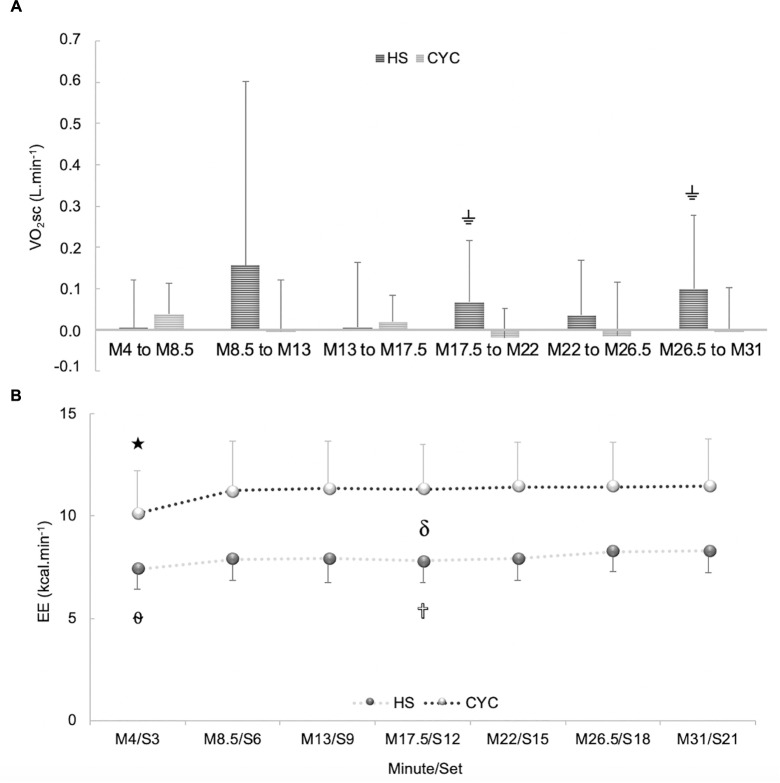
Differences between half-squat (HS) exercise and cycle ergometer (CYC) during constant-load test at each checkpoint: **(A)** Slow component of oxygen uptake (VO_2_sc). **(B)** Energy expenditure (EE). 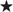 Significantly different from M8.5, M13, M17.5, M22, M26.5, and M31 (*p* < 0.001). 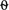 Significantly different from S6, S15, S18, and S21. 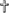 Significantly different from S21. δ Significant differences between cycle ergometer and HS exercise at each checkpoint (*p* < 0.001). 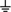 Significantly different from cycle ergometer (*p* < 0.05).

In VO_2_sc at each checkpoint, a significant exercise mode × time interaction effect was observed [*p* = 0.027, *F*_(6,114)_ = 2.48, $\eta_{p}^{2}$ = 0.11, SP = 0.82], along with a significant time effect [*p* < 0.001, *F*_(6,114)_ = 10.61, $\eta_{p}^{2}$ = 0.35, SP = 1.00]. Bonferroni adjustment of multiple comparisons confirmed a greater VO_2_sc in HS than in cycle ergometer testing at the end of exercise (M22/S15, M31/S21) (*p* < 0.05) (Figure 4A).

In EE, a significant exercise mode × time interaction effect was discovered [*p* = 0.001, *F*_(6,120)_ = 3.96, $\eta_{p}^{2}$ = 0.17, SP = 0.97]. A significant time effect [*p* < 0.001, *F*_(6,120)_ = 27.10, $\eta_{p}^{2}$ = 0.58, SP = 1.00] and exercise mode effect were identified [*p* < 0.001, *F*_(6,120)_ = 34.25, $\eta_{p}^{2}$ = 0.63, SP = 1.00]. Bonferroni *post hoc* analysis confirmed a higher EE in cycle ergometer than HS exercise at each checkpoint (*p* < 0.001). A slight and continued EE increase was detected from S3 in HS exercise (*p* < 0.05). A stable EE was observed from M4 in cycle ergometer (*p* > 0.05) (Figure 4B).

**FIGURE 5:**
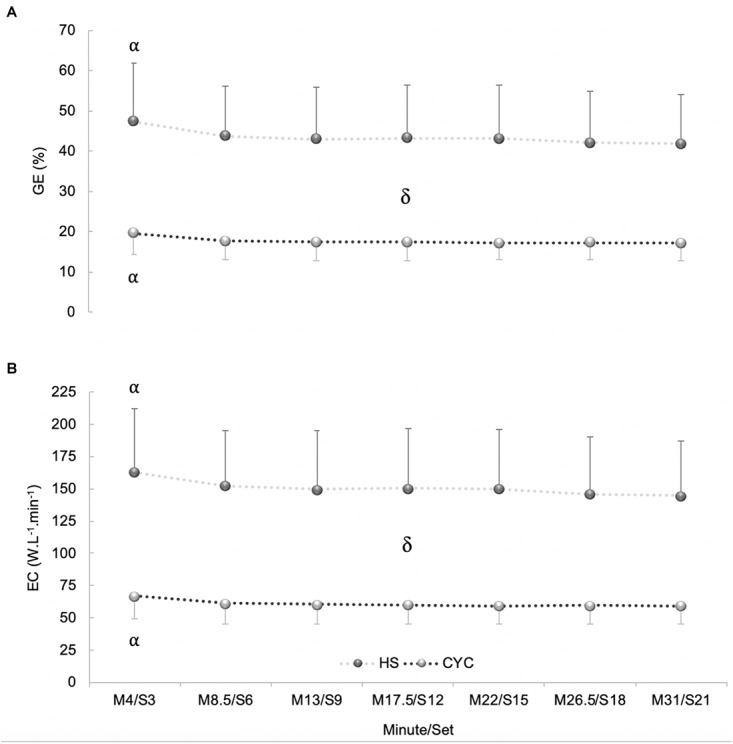
Differences between half-squat (HS) exercise and cycle ergometer (CYC) during constant-load test at each checkpoint: **(A)** Gross efficiency (GE). **(B)** Economy (EC). αSignificantly different from M8.5/S6, M13/S9, M17.5/S12, M22/S15, M26.5/S18, and M31/S21 (*p* < 0.05). δSignificant differences between cycle ergometer and HS exercise at each checkpoint (*p* < 0.001).

In GE, a significant exercise mode x time interaction effect was discovered [*p* = 0.005, *F*_(6,120)_ = 3.31, $\eta_{p}^{2}$ = 0.14, SP = 0.92]. In addition, a significant exercise mode and time effect was found [*p* < 0.001, *F*_(1,20)_ = 61.41, $\eta_{p}^{2}$ = 0.75, SP = 1.00; *p* < 0.001, *F*_(6,120)_ = 22.65, $\eta_{p}^{2}$ = 0.53, SP = 1.00, respectively]. After Bonferroni multiple comparisons, a higher GE was perceived in HS than in cycle ergometer exercise (*p* < 0.001). There were significant differences between M4/S3 vs. all checkpoints in both exercises (*p* < 0.05). However, a non-significant but sustained decrease was produced from M4/S3 in both exercise modalities (∼13%) during constant-load tests (*p* > 0.05) (Figure 5A).

In economy, a significant exercise mode x time interaction effect was found [*p* = 0.013, *F*_(6,120)_ = 2.85, $\eta_{p}^{2}$ = 0.13, SP = 0.88]. A significant exercise mode and time effect was found [*p* < 0.001, *F*_(1,20)_ = 61.66, $\eta_{p}^{2}$ = 0.76, SP = 1.00; *p* < 0.001, *F*_(6,120)_ = 18.84, $\eta_{p}^{2}$ = 0.49, SP = 1.00, respectively]. Bonferroni test determined a higher economy in HS than in cycle ergometer exercise (*p* < 0.001). There were significant differences between M4/S3 vs. all checkpoints in both exercises (*p* < 0.05). However, a non-significant but continued decrease was observed from M4/S3 in both exercise modalities (*p* > 0.05) (Figure 5B).

### Lower Limbs Fatigue

**FIGURE 6:**
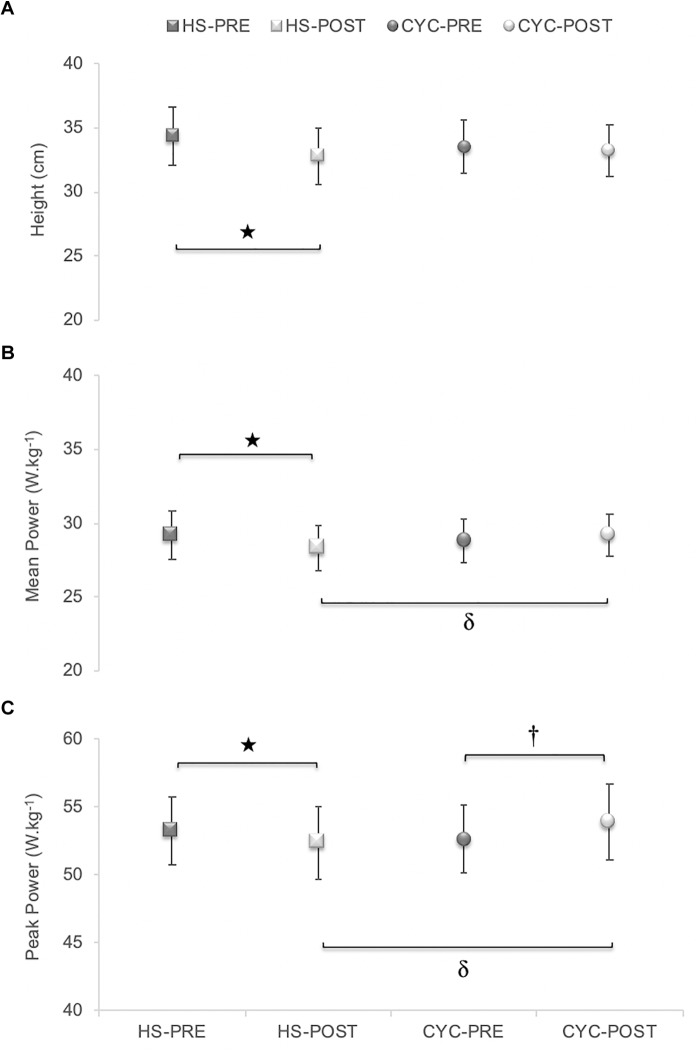
Mechanical fatigue evaluated before and after constant-load exercises using a counter-movement jump test. 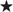
Significant differences between pre- and post-test in HS exercise (*p* < 0.01). ^†^Significant differences between pre- and post-test in cycling exercise (*p* = 0.011). δSignificant differences between cycling and HS tests (*p* < 0.05).

In CMJ test, a significant exercise mode x time interaction effect was observed in jump height [*p* = 0.004, *F*_(1,20)_ = 10.76, $\eta_{p}^{2}$ = 0.35, SP = 0.88], mean power [*p* = 0.003, *F*_(1,20)_ = 11.82, $\eta_{p}^{2}$ = 0.37, SP = 0.91], and peak power [*p* < 0.001, *F*_(1,20)_ = 23.61, $\eta_{p}^{2}$ = 0.54, SP = 0.99]. In Bonferroni test, significant losses were produced between pre- and post-test in jump height (*p* < 0.001), mean power (*p* = 0.001), and peak power (*p* < 0.010) only in HS exercise. Peak power was increased after cycle ergometer test (*p* < 0.05) (Figure 6).

## Discussion

In support of our initial hypothesis, the main novel finding of this study was that the VO_2_sc and EE increased slowly only in HS constant-load test at LT_1_ intensity. As expected, during cycle-ergometer exercise at a constant work rate, a steady-state in pulmonary VO_2_ and EE was reached. These outcomes could justify, at least in part, a decrease observed in jump performance (height and power) only after HS test. Contrary to our expectation, GE/economy in HS exercise did not reduce to a greater magnitude than in cycle ergometer test at the same LT_1_ intensity. In addition, there was a higher response in VO_2_ and heart rate during the constant-load test in cycle ergometer than in the HS exercise.

The results of VO_2_sc obtained in HS exercise (absolute values 141.4 mL in 28 min, relative values 5.05 mL.min^-1^) were slightly higher than in the cycling test (absolute values 131.8 mL in 28 min, relative values 4.7 mL.min^-1^). HS results were reinforced by our previous investigations (Garnacho-Castaño et al., 2018a) that found similar VO_2_sc values in HS exercise (absolute values 153.8 mL in 28 min, relative values 5.49 mL.min^-1^), slightly higher in absolute values (130 mL in 17 min) and slower in relative values (7.6 mL.min^-1^) than that obtained by professional cyclists at intensities clearly above the LT_1_ (80% VO_2max_) (Lucía et al., 2000). These results visibly differed from those reported in well-trained triathletes during constant work rate at 90% of VO_2max_ in cycling (absolute values 269 mL in 10 min 35 s, relative values ∼25 mL.min^-1^) and running (absolute values 21 mL in 10 min 54 s, ∼2 mL.min^-1^) (Billat et al., 1998). These variances of VO_2_sc are not fully understood, though they could be related to the difference in the magnitude of VO_2_sc between exercise modes and load intensity (Carter et al., 2000; Koppo et al., 2002), training status (Burnley and Jones, 2007), and prolonged constant-load tests (Hopker et al., 2017).

The physiological mechanisms that cause the increase of VO_2_sc during constant-load HS test are uncertain because the power output or load equivalent to the LT_1_ intensity means, in theory, the highest power output or load that will not elicit VO_2_sc (Burnley and Jones, 2007). The VO_2_ kinetics observed during constant-load cycling test justified a steady state in VO_2_ and EE at the LT_1_ intensity. In consequence, the blood lactate increased above the resting values, but did not accumulate over time as occurred during both constant-load tests (Garnacho-Castaño et al., 2015a). If VO_2_ continued to increase, especially at the end of the constant-load HS test, it could be assumed that the VO_2_sc is associated with fatigue and a decrease in muscular efficiency, so blood lactate should accumulate at a constant or increasing rate in response to the transition toward a predominantly anaerobic metabolism (O’Connell et al., 2017). The only hypothesis that was confirmed was an increase in VO_2_sc and EE linked to lower limbs fatigue at the end of the HS test. Blood lactate was oxidized in a mainly aerobic metabolism and exercise intensity was considered as being at or below the anaerobic or LT (Svedahl and MacIntosh, 2003).

This detected response of VO_2_sc in HS exercise usually occurs at intensities above the LT_1_ in endurance exercises. Unlike the cycle ergometer test, performing 31 min (21 sets) of HS exercise at the LT_1_ intensity would only be conceivable with a recovery time between each set. Although break durations of 30 s have indicated negligible effects on lactate kinetics during discontinuous protocols (Gullstrand et al., 1994), our HS protocol caused a relative lack of O_2_ supply to muscle loci, further suggesting that an important percentage of the energy derived from anaerobic metabolism might not be quantified by measuring metabolic gas exchange (Garnacho-Castaño et al., 2018a). The HS test probably stimulated a release of anaerobic sources to EE (Tesch et al., 1986) that make it impossible to use steady-state VO_2_ to exactly estimate the EE (Scott et al., 2011).

Another feasible mechanism that would help to better understand the etiology of the VO_2_sc in resistance exercises links the slight increase in pulmonary VO_2_ with the VO_2_ rise into the muscle. It has been suggested that increased leg VO_2_ could explain for ∼85% of the rise in pulmonary VO_2_ (Poole et al., 1991). Probably, VO_2_sc discovered in HS exercise increased leg VO_2_ within the active muscle to a greater magnitude than in the cycle ergometer test and, consequently, EE was augmented only during HS test. The VO_2_sc and EE increase would presumably be associated with an increased ATP cost of force production and or increased O_2_ cost of ATP resynthesis (Cannon et al., 2014; Korzeniewski and Zoladz, 2015). This energy mechanism would force a delayed recruitment of larger and less efficient motor units from the oxidative point of view to compensate the production of attenuated force in those already active motor units. So, a preferential glycogen depletion of the type I fibers (Vøllestad and Blom, 1985) and the recruitment of type II fibers (Whipp, 1994; Barstow et al., 1996) has been postulated as the most acceptable explanation for the VO_2_sc (Gaesser and Poole, 1996).

Glycogen depletion patterns have been detected in type I/II fibers, confirming that both fast-twitch glycolytic muscle fibers and slow-twitch oxidative muscle fibers were activated during high intensity cycling exercise at 80% of VO_2max_. When cycling exercise was performed at moderate intensity (50% of VO_2max_), only type I fibers were recruited and no VO_2_sc was observed (Krustrup et al., 2004). These findings suggest the recruitment of type I fibers by this mechanism probably occurred during constant-load cycling test. For this reason, VO_2_ was not increased and lower limbs fatigue was not induced at the end of constant-power output cycling. The goal of this study was not to evaluate the gradual fibers-type recruitment associated with the energetic cost; therefore, our arguments are based on findings of others. Nevertheless, it can be assumed that VO_2_sc and the corresponding increase in EE could be related to progressive fatigue in HS exercise (Garnacho-Castaño et al., 2018a).

In theory, the recruitment forced of type II fibers should induce higher blood lactate concentrations in HS exercise. We suspect that there was no increase in blood lactate levels because a recovery time of 1 min was established between sets. Beneke et al. (2003) demonstrated that repetitive interruptions (90 s after every 5th minute) during 30 min of constant-load testing decreased blood lactate concentrations to a greater extent than without interruptions. During the rest time between sets, the VO_2_ of the whole body is still raised as a result of elevated post-exercise VO_2_ while the glycolytic rate of the working muscle mass is diminished. Therefore, the rate of lactate removal is directly linked to VO_2_ under the saturation conditions of the substrate.

In addition, the RER was similar and remained stable throughout the constant-load tests despite the VO_2_ was higher in the cycle ergometer than HS exercise. The RER during a constant-load test determines the percentage of carbohydrates and fats that are being used as an energy substrate. In endurance exercises have been observed that the fat oxidation is greater during running on treadmill than in the cycle ergometer at the same relative intensity (Achten et al., 2003; Cheneviere et al., 2010). This variance is partly originated by a greater degree of localized intramuscular tension during cycle exercise, which increases the recruitment of fast-twitch motor units (Carter et al., 2000) which mainly depend on carbohydrates as a fuel substrate. As the exercise intensity increases, the change in substrate metabolism toward greater carbohydrates dependence is related to a higher recruitment of the fast-twitch motor units (Coyle, 2000) and the appearance of free fatty acid entrapment (Romijn et al., 1993).

Maybe these physiological mechanisms occurred, at least in part, in the HS exercise. Probably, the HS exercise caused a higher intramuscular tension per muscular unit in the knee extensors than the cycle ergometer, intensified by the negative or eccentric work of the HS exercise. This mechanism might induce a gradual recruitment of less efficient type II muscle fibers as the initially recruited type I fibers become fatigued (Carter et al., 2000). In preliminary tests, we discovered that a recovery time between series equal to or less than 45 s produced an exponential increase in blood lactate levels and relevant muscular fatigue. The rest time of 1 min accumulated between sets throughout the constant-load HS test was a key factor to prevent a greater increase in the carbohydrates and replenish energy substrates and, therefore, for maintaining blood lactate levels in a stable aerobic metabolism.

Although the total time of the tests was the same in both exercises, the real time of execution was 10 min 30 s in HS exercise. The 20 min 30 s of recovery time during HS test could justify, at least in part, that the VO_2_ and heart rate was lower in the HS test than cycle ergometer exercise. At the muscular level, probably, the HS exercise was more intense, producing a higher local muscular fatigue. Maybe for this reason, greater fatigue was found in lower limbs after the constant-load HS test. It could be deduced that the muscular fatigue produced in HS exercise stimulated the VO_2_sc to a greater extent than the cycle ergometer having a higher cardioventilatory response. Despite these physiological mechanisms, the RPE was the same in both exercises.

In order to explain the VO_2_sc phenomenon, GE was compared in both HS and cycling exercises. GE values in the cycle ergometer test were similar to that obtained by well-trained cyclists (∼18%) during long-term constant-load tests at moderate intensity. In HS exercise, we verify our previous findings with GE values of ∼44%. Values of ∼24–26% have been proposed in professional riders at the power outputs eliciting the LT and the respiratory compensation point during a ramp test (Lucía et al., 2002). Other studies have found lower GE values of 14–16% in world-class sprint cross-country skiers (Sandbakk et al., 2010). These values confirm the idea that GE is conditioned by the exercise modality.

According to results obtained in VO_2_sc and EE during HS exercise, one could expect to discover a greater GE/economy loss throughout the constant-load HS test. Conversely, a 13% loss (non-significant) in GE was observed in both exercise modalities during constant-load tests. Previous studies have demonstrated that GE continues to diminish during prolonged constant-load tests in cycling (Hopker et al., 2017) and HS exercises (Garnacho-Castaño et al., 2018a) at moderate intensity. We suspect that the higher values and the non-loss of GE throughout the constant-load HS test in comparison with the cycle ergometer test were mainly due to the type of muscular action involved in both exercise modalities. HS execution is characterized by eccentric and concentric muscle actions; cycling prioritizes concentric muscle actions (Ericson et al., 1985). A greater increase in O_2_ cost has been shown in no-rebound squats compared to eccentric-concentric squats, and rebound squats stimulate higher efficiency than only concentric squats (Villagra et al., 1993). Pre-stretch allows for storage of elastic energy in the elastic components (muscles and tendons), producing an extra energy that is released during the shortening cycle, probably decreasing O_2_ cost. Furthermore, previous studies have demonstrated higher VO_2_sc in cycling, compared to running (Carter et al., 2000). The authors speculated that the differences between the two exercise modalities were produced by the greater intramuscular tension induced during heavy cycling exercise and the higher eccentric muscle activity in running. This might cause a relatively lower recruitment of the less efficient type II muscle fibers in running (Carter et al., 2000). The pre-stretch could help to prevent a higher VO_2_sc, decreasing O_2_ cost and increasing efficiency in HS exercise to a greater extent than concentric pedaling, avoiding a higher recruitment of type II fibers. Furthermore, the eccentric phase has been demonstrated to be a key factor for improving concentric kinetic/kinematic performance during resistance exercises (Garnacho-Castaño et al., 2018b). Our results demonstrated higher power output levels and a lower VO_2_ during constant-load HS exercise than in the cycling test. This increased power output contributed to improve GE in HS exercise. In consequence, variances in power output measures between a cycle ergometer and a linear position transducer should be considered.

We think that the muscle mass involved during exercise is another factor to consider. Several studies have shown a slower VO_2_sc in running than in cycling (Billat et al., 1998; Carter et al., 2000), or a higher relative increase in VO_2_ per unit of time during arm exercise than in a cycling test (Koppo et al., 2002) when a lower muscle mass was involved or when exercise was focused on a specific muscle group. Although the muscle groups involved in HS and cycling exercises are mainly the knee extensors, during HS exercise other muscle groups (i.e., CORE, back, etc.) are likely activated more than in the cycle ergometer exercise. The greater muscle mass involved may help to increase the whole-body efficiency, diminishing O_2_ cost.

There are some limitations in this study which should be considered. Eccentric muscle action is linked to significantly higher muscle temperatures than concentric muscle action when both are performed at a comparable power output, rate of oxygen uptake or heat production (Nielsen et al., 1972; Pahud et al., 1980). This fact may *per se* increase the metabolic rate without any other additional perturbations of the muscular milieu. This increased temperature during negative work in HS exercise could have altered the VO_2_ kinetics by accelerating the rate-limiting metabolic reaction connected with oxidative phosphorylation and, moreover, accelerating a greater VO_2_ delivery to the capillaries and mitochondria (Koga et al., 1997). It would have been interesting to evaluate how it affects the temperature and the positive (concentric) and negative (eccentric) work at the O_2_ cost and consequently to the VO_2_sc during constant-load tests.

In addition, the different methodology and protocols applied in both exercises during the incremental tests generates some controversy in the location of the LT_1_. This factor could condition the cardioventilatory and metabolic responses during the constant-load tests at LT_1_ intensity, producing a bias when comparing both exercises. However, the results reported during the incremental test (Table 1) revealed that the detection of the LT_1_ in both exercises could occur in an equivalent metabolic instant and a similar exercise intensity. This idea is based on the fact that no significant differences were found in VO_2_, heart rate, blood lactate concentrations and RPE between the HS and the cycle ergometer at the LT_1_. Our findings are supported by the criteria established in a previous study (Binder et al., 2008). In both exercises, LT_1_ occurred at a heart rate of ∼65–70% of the maximum heart rate, a rating of perceived exertion of ∼10 and a blood lactate concentrations of ∼2 mmol.L^-1^, which is considered as a light intensity according to the criterion defined at the time of the LT_1_.

Although it appears that the LT_1_ occurred at a similar metabolic moment and intensity during both incremental tests, the cardioventilatory response during the constant-load test at LT_1_ intensity was lower in HS exercise. The controversy is now focused on knowing whether both constant-load protocols occurred at the same relative intensity (% VO_2max_). To solve this problem, both incremental protocols should have been carried out until exhaustion to determine the VO_2max_ and calculate the percentage of VO_2_ in both constant-load tests. The response of blood lactate levels and RPE observed throughout the constant-load test determined, at least, a predominantly aerobic metabolic intensity.

Finally, several studies (Carter et al., 2002; Koppo et al., 2002) have compared the ventilatory responses and the VO_2_sc between several exercises at the same relative intensity (at, above, below of LT_1_). The behavior of VO_2_ and VO_2_sc is exercise- and intensity-dependent despite they are tested at the same relative or metabolic intensity. Resistance training is typically anaerobic in nature. We think that the most important contribution of this study is that resistance exercises might acquire aerobic metabolic properties selecting a suitable load and manipulating the recovery and execution time of the sets.

## Conclusion

Although the VO_2_ and heart rate responses were higher in cycling exercise, the HS constant-load test induced a greater VO_2_sc and EE than the cycling test at the LT_1_ intensity. GE could benefit from the eccentric phase of the HS exercise. Resistance training conducted at a load intensity equivalent to a predominantly aerobic metabolism could improve local muscular resistance and whole-body efficiency. Thus, relevant implications for both performance and health exercise programs could be considered. This would allow a faster recovery of the muscle groups from one session to another. In the fitness programs, this methodology would help complement the aerobic endurance training with resistance exercises that involve a greater muscle mass (CORE, upper limbs, stabilizers, etc.) and a higher mechanical efficiency in a metabolism that is primarily aerobic. Future research should focus on continuous protocols (without rest periods) as in endurance exercise, combining resistance exercises in the form of circuit training. This scientific knowledge could be an important advance in the assessment of resistance exercises for sports performance and health promotion.

## Data Availability

All datasets generated for this study are included in the manuscript and/or the supplementary files.

## Author Contributions

JM-M and MG-C conceived and designed the research. All authors performed the test protocols and edited, revised, and approved the final version of the article. MG-C and JM-M analyzed the data. LA-A, NS-P, MB, RF-R, LC, and EC contributed reagents, materials, and analysis tools. LA-A, JM-M, RF-R, and MG-C prepared the figures and drafted the article.

## Conflict of Interest Statement

The authors declare that the research was conducted in the absence of any commercial or financial relationships that could be construed as a potential conflict of interest.
